# Parent–infant closeness after preterm birth and depressive symptoms: A longitudinal study

**DOI:** 10.3389/fpsyg.2022.906531

**Published:** 2022-07-22

**Authors:** Liisa Lehtonen, Siri Lilliesköld, Kris De Coen, Liis Toome, Ana Gimeno, Sylvia Caballero, Rasa Tameliene, Sabine Laroche, Jana Retpap, Hege Grundt, Marie-Rose Van Hoestenberghe, Caryl Skene, Bernd Pape, Anna Axelin, on behalf of the Separation, Closeness Experiences in Neonatal Environment (SCENE) research group

**Affiliations:** ^1^Department of Pediatrics and Adolescent Medicine, Turku University Hospital, Turku, Finland; ^2^Department of Clinical Medicine, University of Turku, Turku, Finland; ^3^Department of Neonatology, Karolinska University Hospital, Stockholm, Sweden; ^4^Department of Women’s and Children’s Health, Karolinska Institutet, Stockholm, Sweden; ^5^Department of Neonatal Intensive Care, Ghent University Hospital, Ghent, Belgium; ^6^Department of Neonatal and Infant Medicine, Tallinn Children’s Hospital, Tallinn, Estonia; ^7^Neonatal Intensive Care Unit, La Fe Hospital, Valencia, Spain; ^8^Department of Neonatology, Hospital General Universitario Gregorio Marañón, Madrid, Spain; ^9^Department of Neonatology, Lithuanian University of Health Sciences, Kaunas, Lithuania; ^10^Neonatal Intensive Care Unit, University Hospital Antwerp, Antwerp, Belgium; ^11^University of Antwerp, Antwerp, Belgium; ^12^Tartu University Hospital, Tartu, Estonia; ^13^Department of Paediatrics, Haukeland University Hospital, Bergen, Norway; ^14^Department of Neonatal Intensive Care, Ziekenhuis Oost-Limburg, Genk, Belgium; ^15^Sheffield Teaching Hospitals NHS Foundation Trust, Sheffield, United Kingdom; ^16^Turku Clinical Research Center, Turku University Hospital, Turku, Finland; ^17^School of Technology and Innovations, University of Vaasa, Vaasa, Finland; ^18^Department of Nursing Science, University of Turku, Turku, Finland; ^19^Department of Women’s and Children’s Health, University of Uppsala, Uppsala, Sweden

**Keywords:** preterm birth, parenting, postpartum depression, skin-to-skin contact, neonatal intensive care unit

## Abstract

**Background:**

Preterm birth increases the risk for postpartum depression in both mothers and fathers, calling for strategies to alleviate and prevent depressive symptoms in parents of preterm infants. The aim of this study was to assess the association between early parent-infant closeness and later depressive symptoms among parents of preterm infants. We hypothesized that longer duration of closeness associate with fewer depressive symptoms in both parents.

**Methods:**

This prospective cohort study included 23 neonatal intensive care units (NICUs) from 15 countries in 2018 to 2020. Each unit recruited families with preterm infants aiming to 30 families. The total duration of parents’ presence in the NICU, and separately parent-infant skin-to-skin contact and holding, were measured using a Closeness Diary up to 14  days. The Edinburgh Postnatal Depression Scale (EPDS) was used at discharge and at 4  months corrected age of the infant.

**Results:**

The study included 684 mothers and 574 fathers. The median presence was 469   min (Q1 258 and Q3 1,087) per 24   h for the mothers and 259   min (Q1 100 and Q3 540) for the fathers; mean EPDS scores were 9.2 (SD 5.0) and 6.3 (SD 4.4) at discharge and 6.6 (4.7) and 4.3 (4.2) at 4  months, respectively. Parents’ presence and depressive symptoms varied greatly between the units. Parents’ presence as the total measure, or skin-to-skin contact and holding separately, did not associate with depressive symptoms in either mothers or fathers at either time point (adjusted).

**Conclusion:**

No association was found between the duration of parent-infant closeness in the neonatal unit and parents’ depressive symptoms. The beneficial effects of family-centered care on parents’ depression seem to be mediated by other elements than parent-infant physical closeness. More research is needed to identify the critical elements which are needed to alleviate parents’ depression after NICU stay.

## Introduction

Preterm birth implies several consequences, such as a risk for abnormal child development and compromised parental wellbeing, including a risk for depression (Vigod et al., 2010; Carson et al., 2015; Helle et al., 2015; Pace et al., 2016). Maternal postpartum depression has been shown to associate with suboptimal socio-emotional and cognitive development of the child (Burger et al., 2020; Morgan et al., 2021). However, even if various interventions have been studied, with mixed quality of methodology, none have proven particularly effective for preventing depression in the parents of preterm infants.

Today, parent-infant closeness, as opposed to separation, is recognized as a key issue in the neonatal intensive care setting when implementing infant- and family-centered care supporting later infant and family health. Closeness can be described both in terms of physical closeness, ranging from the parents’ presence in the unit to being in direct skin-to-skin contact with the infant, as well as in terms of emotional closeness, experiencing feelings of love and affection (Flacking et al., 2012). A wide variation has previously been reported in the amount of parent-infant physical closeness occurring in neonatal intensive care units (NICUs; Raiskila et al., 2017), which may reflect variations in social policies (e.g., the length of parental leave) and unit policies and design, as well as the care culture within the unit regarding support to parent-infant closeness. Skin-to-skin contact is recommended as the best practice to promote closeness in the NICU and can, in most cases, be started safely shortly after birth (Nyqvist et al., 2010).

There are a number of potential pathways through which physical closeness may affect postpartum depressive symptoms in the parents of preterm infants. The postpartum period is a sensitive period for both mothers and fathers to develop a loving bond to their infant, strongly regulated by oxytocin hormone and its effects in the limbic network of the brain, the center for reward and emotionality (Feldman, 2016). Skin-to-skin contact increases parents’ oxytocin levels (Vittner et al., 2019). The smell of the infant and parenting experiences have also been shown to elicit structural and functional neurobiological changes in the subcortical brain areas of the parent, including the medial preoptic area of the hypothalamus, the hippocampus, amygdala and dopamine reward circuit, and their cortical connections (Abraham et al., 2018). Parent’s physical presence is required for skin-to-skin contact, olfactory stimuli and early parenting experiences. Therefore, a delay of parents’ presence in the NICU following preterm birth disrupts the early processes of bonding and parenting and, consequently, might increase depressive symptoms.

A systematic review and meta-analysis (Scime et al., 2019) showed small, but significant, effects of skin-to-skin contact on postpartum depressive symptoms among mothers of preterm infants. In mothers of very preterm infants, skin-to-skin contact in the delivery room was shown to decrease depression on the third postpartum day but the effect was not seen at discharge (Mehler et al., 2020). Skin-to-skin contact applied post-discharge decreased depression in mothers of low-birth-weight infants in a large, randomized study in India (Sinha et al., 2021). Separation from the infant has been described as the most difficult aspect for parents when their newborn infant is in a NICU (Wigert et al., 2006; Obeidat et al., 2009), leading the parents to feel disconnected from their newborn and incompetent in their parenting role (Sisson et al., 2015; Spinelli et al., 2016). Parental role attainment among parents of preterm infants may have an intricate relationship with postpartum depression. Stress caused by parental role alteration is shown to be a psychological risk factor (Rogers et al., 2013). An educational intervention for the staff, Close Collaboration with Parents, decreased the depressive symptoms of mothers of preterm infants discharged from the unit which carried out the program. The program facilitates staff abilities to involve parents in the care of their newborn from birth and to support the parent-infant relationship. Maternal depressive symptoms remained lower at 4 to 6 months and 2 years after the due date (Ahlqvist-Björkroth et al., 2019, 2022). As the same intervention was also shown to increase parent-infant physical closeness in nine hospitals (He et al., 2021), parent-infant closeness can be expected to be one of the mechanisms preventing depressive symptoms.

Currently, we do not have knowledge about how different types of early parent-infant closeness influence parents’ depressive symptoms. It is noteworthy that studies including fathers are rare. Thus, the aim of this prospective study was to assess the association between early parent-infant physical closeness and later depressive symptoms among both mothers and fathers in a large international group of NICUs. We hypothesized that a longer duration of parents’ presence, especially skin-to-skin contact and holding, would associate with fewer depressive symptoms in parents.

## Materials and methods

### Study design and participants

This study question was one of the two main aims of the 2nd International Closeness Survey. The other aim was to study the association between the quality of family-centered care in the units and later parental depression (Axelin et al., 2021). STROBE guidelines were followed in reporting the study. The study included 23 NICUs from 15 countries: Australia, Belgium, Canada, Croatia, Denmark, England, Estonia, Finland, Iceland, Lithuania, the Netherlands, Norway, Poland, Spain and Sweden. Data on parent-infant closeness and the parents’ depressive symptoms were collected prospectively, aiming to include 30 families from each unit. The data collection was carried out between March 2018 and February 2020, lasting from 2 to 12 months in each unit.

The families were invited to participate in the study if their infant was born below 35 weeks of gestation, admitted in the participating NICU, and the family had a mobile phone for data collection for another arm of this study. The exclusion criteria included: (1) the infant’s estimated hospital stay length being less than 3 days, (2) any life-threatening medical condition of the infant, (3) triplets, and (4) parents who were unable to read English or the local language at the study site. Recruitment took place as soon as appropriate before the sixth day of life, except for 2 units where the local ethics committee did not permit recruitment until the second week of life. The study units kept a record of all admissions of infants below 35 gestational weeks in order to evaluate the representativeness of the study participants. The data included the infants’ gestational age, birthweight, length of hospital stay, and the distance from home to hospital (as driving time) as well as the reasons of ineligibility if present.

### Measurements

A Closeness Diary (Axelin et al., 2020) was used to assess the mothers’ and fathers’ presence in the neonatal unit as the total measure of closeness and separately mother-infant and father-infant holding and skin-to-skin contact (mutually exclusive) during first weeks after the birth. The diary was asked to be filled in by the parents for 14 days, or until discharge if earlier. The diary was a paper diary, and it was kept at bedside. With five-minute accuracy, the parents drew a timeline showing when they were present in the unit, held the infant and provided skin-to-skin care. Durations of presence, holding and skin-to-skin contact were calculated as minutes per day. Skin-to-skin contact referred to the time the baby spent without clothes on the bare chest of the parent; holding referred to the time when the baby was held with clothes on by the parent. Separate slots were assigned for the mothers’ and fathers’ presence, holding and skin-to-skin contact. The parents were asked at the beginning of the study about family background characteristic and the timing of the initial contact with their infant. They were asked how many hours or days after birth they (1) saw their baby for the first time, (2) held their baby for the first time, and (3) had the first skin-to-skin contact with their baby.

The Edinburgh Postnatal Depression Scale (EPDS; Cox et al., 1987) was used to assess depressive symptoms in both mothers and fathers when the infant was discharged from hospital and again 4 months after the due date. The EPDS is a widely used and validated self-report screener, assessing the severity of 10 emotional depressive symptoms during the past 7 days. The original EPDS scoring system as developed by Cox has been translated and validated in many languages and has become a popular screening measure for postnatal depressive symptoms. For Croatian, Estonian, Finnish and Polish units no validated EPDS were available; English EPDS was translated following guidelines for translation and cultural adaptation of research questionnaires (Wild et al., 2005). Mothers having a score of 13 or more and fathers having a score of 10 or more were considered to be in a risk for having depression. These are validated cut-off scores used to screen for major depression (Matthey et al., 2006; Edmondson et al., 2010). An additional analysis was run by using a cut-off value of 10 for the mothers as well.

### Statistical analysis

The association between the amount of parent-infant closeness (presence, holding, and skin-to-skin contact) and parent depressive symptoms was assessed using separate linear mixed models for repeated measures with units as random effects applying compound symmetry covariance structure. The depression scores were square root transformed prior to analysis, as this was necessary in order to meet the residual normality requirement in such models.

All analyses were adjusted for the infants’ gestational age, multiple status (singleton/twin), maternal education (divided into four categories: primary education; high school or vocational education; bachelors’ degree; master’s or doctor’s degree), relationship status (single/living in relationship), and sibling status (older siblings present/no siblings). Also the closeness x time interactions and the interactions of the confounders with time were included in order to allow for different degrees of association at discharge and at 4 months corrected age.

The analyses were carried out using SAS for Windows version 9.4 (SAS Institute Inc., Cary, NC, United States). Values of *p* below 0.05 were considered statistically significant.

### Ethical considerations

The study was approved by the Ethics Committee of the Hospital District of Southwest Finland as well as by the scientific research committee of the Pediatric Department of the Turku University Hospital, Finland (T08/011/18). In addition, each study site sought approval from their local research ethics board and/or hospital as required. Data collection began at each site after local approval was obtained. Eligible parents were given oral and written information about the study procedures, and those who agreed to participate provided written informed consent. Study questionnaires and documents were identified with an identification code to conceal the participants’ identities. The electronic platform REDCap used in this study required passwords for access.

## Results

### Participants

**Figure 1:**
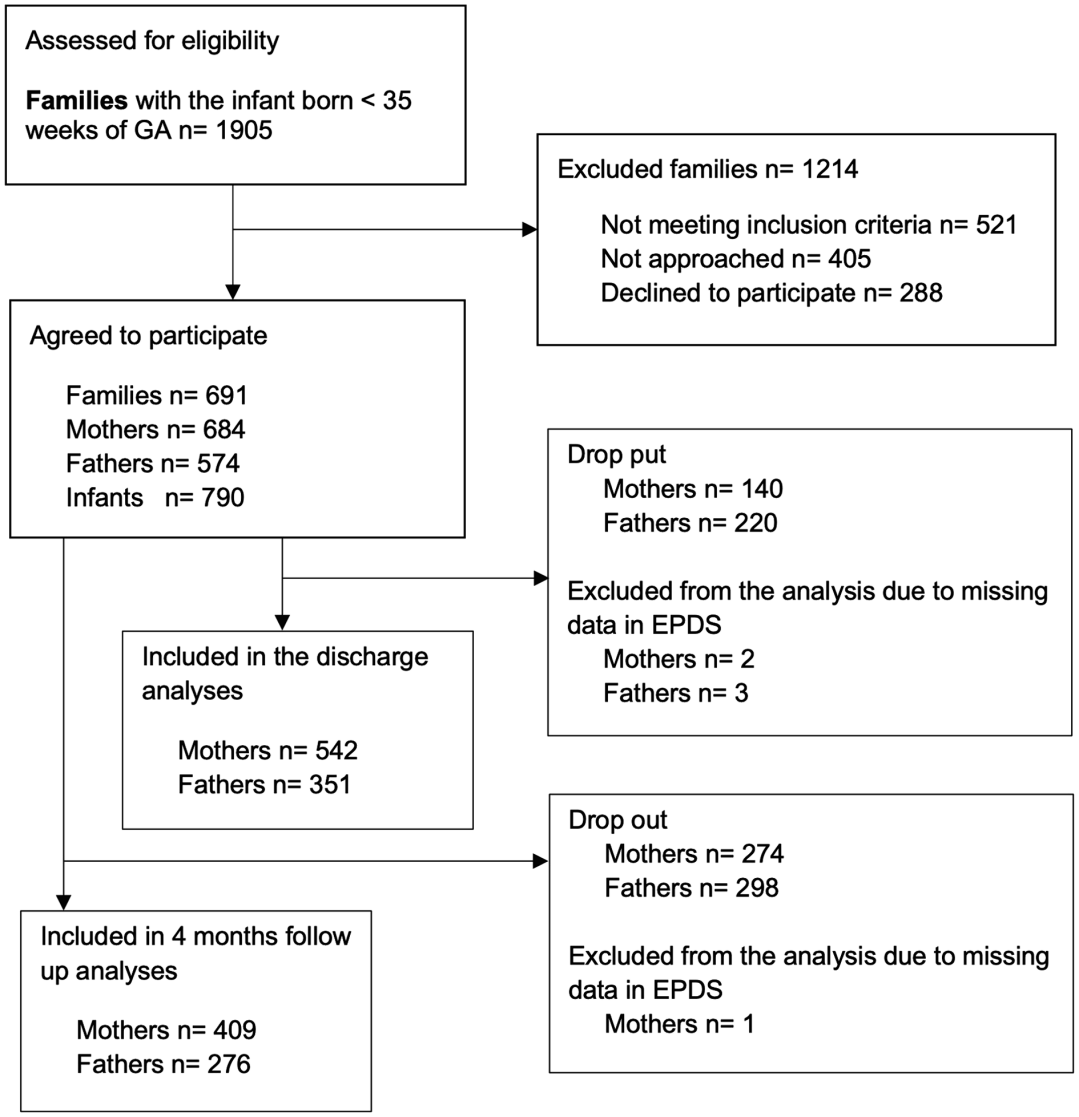
Flowchart on eligible families, those approached and those who agreed to participate.

The study participants included 684 mothers and 574 fathers. The flowchart (Figure 1) shows the eligible families, those approached and those who agreed to participate. Parent-infant closeness data and data on depressive symptoms were available from 542 mothers and 351 fathers at discharge and 409 mothers and 276 fathers at 4 months after the due date. The comparison of the group with responses at both follow up points and the group with partial or no responses are shown in Supplementary Table S1. There was a total of 660 infants in the final study group. Their diary data collection started on day of life 4.28 (mean, SD 5.21) and the diaries were filled for 11.6 (mean, SD 3.62) days. The mothers saw their infant at median of 1 (Q1 0–Q3 5) hours and fathers at 0 (0–1) hours after the birth; the mothers held their infant for the first time at median of 24 (Q1 3–Q3 72) hours and the fathers at 24 (4–96) hours after the birth; the first skin-to-skin contact with the mother started at median of 24 (Q1 6–Q3 72) hours and with the fathers at 48 (15–120) hours after the birth. The other background characteristics of the families included in the final analyses are shown in Tables 1, 2.

**Table 1:** The background characteristics of the families included in the final analysis.

Variable	Mother (*n* = 565)	Father (*n* = 393)
Age (years) median (*Q1–Q3*)	*n* = 564	*n* = 390
	31.0 (28.0–35.0)	33.0 (30.0–37.0)
Education *n* (%)	*n* = 552	*n* = 380
Primary education	28 (5.1)	22 (5.8)
Second level	227 (41.1)	166 (43.7)
Bachelor’s degree	106 (19.2)	66 (17.4)
Master’s/doctor’s degree	191 (34.6)	126 (33.2)
Occupation *n* (%)	*n* = 562	*n* = 387
Paid work	470 (83.6)	370 (95.6)
Unemployed	35 (6.2)	12 (3.1)
Student	18 (3.2)	3 (0.8)
Home maker	38 (6.8)	2 (0.5)
Native language *n* (%)	*n* = 563	*n* = 390
Native	499 (88.6)	359 (92.1)
Other	64 (11.4)	31 (7.9)
Smoking *n* (%)	*n* = 561 31 (5.5)	*n* = 386 66 (17.1)
Relationship *n* (%)	*n* = 560	*n* = 391
Single	18 (3.2)	1 (0.3)
In relationship, not cohabiting	34 (6.1)	20 (5.1)
In relationship, cohabiting	508 (90.7)	370 (94.6)
Siblings at home *n* (%)	*n* = 553	*n* = 385
Yes	242 (43.8)	149 (38.7)
No	311 (56.2)	236 (61.3)

**Table 2:** Infant background characteristics and the initiation and length of diary data collection in the final study group as a whole and the lowest and highest means/medians of the units.

Infant characteristics	*n* = 660	Range of the unit means/medians
Gestational age (weeks) median (*Q1–Q3*)	31.9 (29.0–33.7)	27.3–33.1
Weight at birth (g) mean (SD)	1,602 (576)	1,053–1,936
Gender *n* (%)		
Female	286 (43.3)	
Male	374 (56.7)	
Delivery *n* (%)		
C-section	375 (56.8)	
Vaginal	285 (43.2)	
Twins *n* (%)		
Singleton	470 (71.2)	
Twins	190 (28.8)	
Age at the initiation of diary data collection (days) mean (SD)	4.28 (5.21)	1.5–18.8
Number of diary days per infant mean (SD)	11.6 (3.62)	9.0–13.3
Length of hospital stay (days) median (*Q1–Q3*) *n* = 658	23 (13–41)	15–48

### Parent–infant closeness

The mothers were present for a mean of 658 (SD 463) minutes, holding the baby for 72 (121) minutes and in skin-to-skin contact with the baby for 106 (122) minutes per day during the diary days; the fathers were present for 427 (383) minutes, holding the baby for 23 (42) minutes and in skin-to-skin contact with the baby for 74 (92) minutes per day. The data is shown on the unit level in Table 3.

**Table 3:** The duration of parent-infant closeness (minutes per 24 h) during the diary days in the 23 units.

	In all units	Shortest duration per unit	Longest duration per unit
Presence *median (Q1–Q3)*			
Mothers	469 (258–1,087)	137 (75–227)	1,367 (1,318–1,410)
Fathers	259 (100–540)	28 (8–75)	1,127 (724–1,283)
Holding *median (Q1–Q3)*			
Mothers	15 (0–85)	0 (0–0)	106 (26–209)
Fathers	0 (0–24)	0 (0–0)	27 (11–88)
Skin-to-skin contact *median (Q1–Q3)*			
Mothers	56 (15–124)	0 (0–0)	235 (164–344)
Fathers	24 (0–82)	0 (0–0)	170 (116–277)

### Early mother–infant closeness and maternal depressive symptoms

The mothers’ pooled mean EPDS score was 9.2 (SD 5.0) at discharge and 6.6 (4.7) at 4 months after the due date. Between the units, their EPDS mean score varied from 5.8 to 12.5 at discharge and 4.7 to 9.6 at 4 months. A total of 25.3% of the mothers exceeded the EPDS threshold at discharge and 12.3% after 4 months. The proportion of mothers exceeding the screening threshold (13 or more) varied from 0 to 53.3% at discharge and 0 to 27.3% at 4 months after the due date between the units.

The duration of the mothers’ presence, holding or skin-to-skin contact did not associate with either the mean EPDS score or the proportion of mothers exceeding the screening threshold at either time point when adjusted for the potential confounders. The results remained unchanged in a post-hoc analysis with a lower threshold of 10. The Pearson correlation coefficients are shown in Table 4.

**Table 4:** Pearson correlations coefficients (r) between the types of parent-infant closeness (minutes per day) and square-rooted EPDS scores at discharge and at 4 months of corrected age of the infant for the mother and fathers.

	At discharge	At 4 months
Mothers		
Presence	−0.117	−0.065
Holding	−0.130	0.000
Skin-to-skin	−0.058	−0.041
Fathers		
Presence	−0.069	−0.034
Holding	−0.054	0.048
Skin-to-skin	−0.005	−0.020

### Early father–infant closeness and paternal depressive symptoms

The fathers’ pooled mean EPDS score was 6.3 (SD 4.4) at discharge and 4.3 (4.2) at 4 months. Their EPDS mean score varied from 3.3 to 7.9 at discharge and 1.6 to 7.5 at 4 months between the units (excluding two centers in which only one father responded at both time points). A total of 8.3% of the fathers exceeded the EPDS threshold at discharge and 5.8% after 4 months. The proportion of fathers exceeding the screening threshold (10 or more) varied from 0 to 23.1% at discharge and 0 to 20.0% at 4 months between the units.

The duration of the fathers’ presence, holding or skin-to-skin contact did not associate with either the mean EPDS score or the proportion of fathers exceeding the screening threshold at either time point when adjusted for the potential confounders.

## Discussion

This large prospective study about parent-infant closeness and parental depression included parents of preterm infants in 15 countries. It found no association between the duration of parents’ presence in the NICU during the first weeks of hospitalization and their later depressive symptoms, neither did the duration of holding or skin-to-skin contact associate with depressive symptoms in mothers or fathers.

We focused on physical closeness between parents and preterm infants in the beginning of their hospital stay, paying attention to the duration of early contact at different degrees of proximity. Although this two-week period covered only a fraction of the whole hospital time for the most immature infants, this timeframe was chosen as it can be argued that the early postpartum period is an important time period for forming a bond between the parent and the infant. This is also a time period in an NICU context that traditionally involves disruption of parent-infant closeness as well as acute parental distress (Lefkowitz et al., 2010).

Depression was measured twice. Measurements at discharge are commonly used, and the differences in early parent-infant closeness could already have made a difference. The second time point was chosen to evaluate prolonged depression with a stronger clinical significance (Korja et al., 2008). The levels of depressive symptoms were higher in mothers than in fathers. This is in alignment with what is found in general population norms (O'Hara and Swain, 1996; Cameron et al., 2016), as well as in previous studies on the parents of preterm infants (Helle et al., 2015; Yoldas et al., 2020). The level of depressive symptoms decreased over time, similarly to earlier studies (Pace et al., 2016). Our findings call attention to the prevention and alleviation of postpartum depression in both the mothers and fathers of preterm infants.

Parenting interventions in the NICU have been shown to have various effects on parental depressive symptoms. Some of them have decreased maternal depression (Melnyk et al., 2006; Ahlqvist-Björkroth et al., 2019), while others have had short-term or less robust effects (Silverstein et al., 2011; Welch et al., 2016), and some did not affect depression (Zelkowitz et al., 2011). A meta-analysis concluded that NICU-based interventions decreased depression (Mendelson et al., 2017). Although some studies have shown fewer depressive symptoms in mothers and fathers if their infant is cared for in a single-family room (Lester et al., 2014; Tandberg et al., 2019), a meta-analysis did not find single-family room design to influence the levels of depressive symptoms (van Veenendaal et al., 2020). Two systematic reviews and a meta-analysis have demonstrated that skin-to-skin interventions are associated with a reduction in maternal depressive symptoms, although it is concluded that further assessment is warranted (Athanasopoulou and Fox, 2014; Scime et al., 2019). In conclusion, it remains unclear which elements of the interventions are effective in supporting the mental health of parents.

Depressive symptoms among the parents of preterm infants varied between the units despite using the same methods, cut-offs, and age points. Depressive symptoms associated with the quality of family-centered care reported by the parents (Axelin et al., 2021). This variation suggests that there are care practices which are more successful in supporting the parents’ psychological well-being than others. Our results in this study suggest that focusing only on parent-infant physical closeness is not sufficient. The perceived support from health care staff, as well as social support provided by the partner and family, might be important mediating factors as well, and have been found to affect postnatal depressive symptoms (Axelin et al., 2021; Wells and Aronson, 2021). Most likely, the different elements are interrelated to each other, with a potential of enhancing each other. Parents might be supported by providing them an active and meaningful role in caretaking; being present is necessary but the quality of that presence might be what is important for their well-being. The Close Collaboration with Parents program, which had favorable effects in mothers’ depression (Ahlqvist-Björkroth et al., 2019), has been shown to impact a wide range of family-centered care practices (Toivonen et al., 2020). Our findings in this study suggest that the other elements of family-centered care might play a more important role than parent-infant physical contact.

The strengths of this study included the large number of participating NICUs, providing us with a large variation in physical closeness. The prospective data collection provided us with detailed, day-to-day data. The data collection tool, Parent-Infant Closeness Diary, has been shown to be a reliable and feasible tool when collecting data on physical parent-infant closeness for up to a 2-week period (Axelin et al., 2020).

There are limitations in this study. Although a vast majority of eligible families participated in this study, it is likely that those with little presence at the hospital were not approached and that those with more depressive symptoms declined participation. Yet, our study included a large variation of parental presence and depressive symptoms and provided us with a representative group of families in the NICU. Due to the unpredictable nature of preterm birth, depression could not be screened for during the prenatal period and, therefore, we have not been able to control for prior depression in our cohort. Furthermore, even if the EPDS is a validated tool with recommended cut-off points for postpartum depression in both mother and fathers, there are cultural differences in the expression of depressive symptoms leading to heterogeneity in sensitivity and specificity for detecting different levels of postnatal depression across different cultural settings (Gibson et al., 2009). For the purpose of this study, however, uniform cut-off thresholds were chosen for the whole cohort and a lower cut-off threshold was used for the fathers than the mothers as recommended in the literature.

In conclusion, our results did not confirm the hypothesis that the duration of parents’ presence in the NICU during the first weeks of hospital care has a major role in the prevention of parental depressive symptoms. Our results suggest that care culture should be studied broadly including several elements of family-centered care to understand the mechanisms behind the variable levels of depressive symptoms among parents in different units.

## Data availability statement

The raw data supporting the conclusions of this article will be made available by the authors, without undue reservation.

## Ethics statement

The studies involving human participants were reviewed and approved by Committee of the Hospital District of Southwest Finland as well as by the Scientific Research Committee of the Pediatric Department of the Turku University Hospital, Finland (T08/011/18). In addition, each study site sought approval from their local research ethics board and/or hospital as required. The patients/participants provided their written informed consent to participate in this study.

## Author contributions

LL and AA conceptualized and designed the study and drafted the initial manuscript together with SLi. BP did the statistical analyses. All authors participated in the data collection and reviewed and revised the manuscript. All authors contributed to the article and approved the submitted version.

## Conflict of interest

The authors declare that the research was conducted in the absence of any commercial or financial relationships that could be construed as a potential conflict of interest.

## Publisher’s note

All claims expressed in this article are solely those of the authors and do not necessarily represent those of their affiliated organizations, or those of the publisher, the editors and the reviewers. Any product that may be evaluated in this article, or claim that may be made by its manufacturer, is not guaranteed or endorsed by the publisher.
