# Supplementary material for: Parent–infant closeness after preterm birth and depressive symptoms: A longitudinal study
Source: Front Psychol. 2022 Jul 22;13:906531. doi: 10.3389/fpsyg.2022.906531 (PMC9551610; doi:10.3389/fpsyg.2022.906531)
Supplement: Supplementary file 1 [file Table_1.docx]

**Supplemental Table S1**. The comparison of the group with responses at both follow up points (discharge and 4 months) called as full response group and the group with partial or no responses in the follow up. Numbers or means (SD) are presented if not otherwise indicated.

| **Mothers** | **Full response group** | **Others** |
| --- | --- | --- |
| Education  Primary education  Second level  Bachelor’s degree  Master’s/doctor’s degree | 14  146  79  142/381 | 14  81  28  49/172 |
| Relationship  Yes | 372/384 (9.6%) | 171/177 (10.0%) |
| Siblings/yes | 152/376 (40.4%) | 90/178 (50.5%) |
| Gestational age, weeks | 31.0 (3.0) | 30.9 (3.0) |
| Distance to home, km | 40.0 (57.5) | 43.2 (63.7) |
| The earliest EPDS | 9.0 (4.9) | 9.0 (5.2) |
| Presence | 680.5 (464) n=386 | 583.3 (454) n=180 |
| SSC | 110.1 (121) n=386 | 93.6 (119) n=180 |
| Holding | 73.7 (111) n=386 | 75.9 (138) n=180 |
| **Fathers** | **Full response group** | **Others** |
| Education  Primary education  Second level  Bachelor’s degree  Master’s/doctor’s degree | 5  70  57  99/231 | 10  61  30  54/155 |
| Relationship  Yes | 233/233 | 160/161 |
| Siblings/yes | 82/230 (35.6%) | 68/158 (43.0%) |
| Gestational age, weeks | 30.8 (3.0) | 30.6 (2.7) |
| Distance to home, km | 37.5 (54.0) | 41.1 (56.7) |
| The earliest EPDS | 6.1 (4.5) | 5.9 (4.3) |
| Presence | 450.2 (383.4) n=234 | 362.1 (366.8) n=162 |
| SSC | 80.8 (91.7) n=234 | 56.7 (86.0) n=162 |
| Holding | 23.8 (42.4) n=234 | 19.1 (39.5) n=162 |
